# The shift in rabies epidemiology in France: time to adjust rabies post-exposure risk assessment

**DOI:** 10.2807/1560-7917.ES.2018.23.39.1700548

**Published:** 2018-09-27

**Authors:** Perrine Parize, Laurent Dacheux, Florence Larrous, Hervé Bourhy, the French network of antirabies clinics

**Affiliations:** 1 Institut Pasteur, Unit Lyssavirus Dynamics and Host Adaptation, National Reference Center for Rabies and WHO Collaborating Centre for Reference and Research on Rabies, Paris, France; 2 The members of the network are listed at the end of the article

**Keywords:** rabies, lyssaviruses, zoonoses, post-exposure prophylaxis, public health policy, vaccine-preventable diseases

## Abstract

The epidemiology of rabies in France and western Europe has changed during the past 22 years. In France, rabies in non-flying terrestrial mammals was declared to be eliminated in 2001, and the risk of rabies is now limited to contact with bats, rabid animals illegally imported from rabies-enzootic countries and traveller exposure in enzootic areas. We analysed the epidemiology of rabies in France from 1995 to 2016, describing and analysing data on human rabies surveillance as well as data on post-exposure prophylaxis (PEP) collected from the network of French antirabies clinics. Over the study period, seven individuals were diagnosed with rabies in France, all of whom were infected outside mainland France. PEP data analysis revealed an expected overall decrease in PEP administration for individuals exposed in mainland France, but there was still overuse of anti-rabies drugs, given the very low epidemiological risk. On the other hand, a significant increase in PEP delivered to individuals exposed abroad was evidenced. These epidemiological trends indicate that clear guidelines should be provided to support physicians’ efforts to adjust rabies risk assessment to the evolution of the epidemiological situation.

## Introduction

Rabies is a zoonotic disease caused by a neurotropic virus of the Lyssavirus genus. The virus is transmitted from animal to human by bite, scratch or by direct exposure of mucosal surfaces to saliva from an infected animal [1]. All mammals are susceptible to rabies, but only a few species are important as reservoirs for the disease (dogs, some other carnivores and bats) [2]. Human-to-human transmission of rabies is rare and mainly reported in the setting of tissue and solid-organ transplantation [3-5]. The rabies virus reaches the brain by centripetal propagation mediated by retrograde transneuronal transfer, and once clinical signs appear the disease almost invariably progresses to fatal encephalitis [6]. The onset of clinical symptoms of rabies and death can be prevented by adequate post-exposure prophylaxis (PEP) including vaccines and, if required, rabies immunoglobulin (RIG) [7,8]. However, rabies still causes tens of thousands of deaths worldwide every year, mostly in the developing world where control measures in dogs are not implemented and the majority of the population do not have access to PEP [9-14].

In western Europe rabies is rare due to its elimination, first in dogs at the beginning of the 20th century, and then progressively in foxes since the 1980s [15]. The last human case of autochthonous rabies in mainland France was reported in 1924 and rabies was officially declared eliminated in non-flying terrestrial mammals in 2001. This status is being maintained by strong regulation measures and rigorous public health management systems [16]. Currently, the risk of autochthonous rabies in France is limited to contact with bats, which have regularly been found to be infected with lyssaviruses, or to contact with rabid animals illegally imported from rabies-enzootic countries (mainly in North Africa) [17-21]. French travellers may also be exposed to rabid animals in enzootic areas outside France [22].

In France, human rabies surveillance is ensured by mandatory notification to the Regional Health Agencies. The National Reference Centre for Rabies (NRCR), is responsible for rabies diagnosis in humans and in the animals responsible for human exposure in all French territory (France includes mainland France and French overseas regions and territories, population 67.2 million on January 2018). The NRCR also annually collates national data concerning PEP collected from an official network of 70 antirabies clinics that are designated by the Directorate General for Health and distributed throughout French territory [23]. There are two approved PEP schedules in France, which consist of a course of four doses of rabies vaccine administered over three visits (Zagreb PEP regimen) or five doses administered over five visits (Essen PEP regimen). RIG is also given, if the exposure is considered to be particularly high-risk. In the absence of specific national rabies prophylaxis guidelines, French antirabies clinics do not apply PEP in a homogenous manner. Clinicians refer either to international guidelines developed mainly for enzootic countries [7,8], or to recommendations from rabies-free areas such as those published recently by Public Health England (PHE) [24] and the conclusions of dedicated working groups [16]. However, the majority of physicians refer to World Health Organization (WHO) guidelines, which recommend PEP for category II exposures (nibbling of uncovered skin, minor scratches or abrasions without bleeding, licks on broken skin) and PEP with RIG for category III exposures (single or multiple transdermal bites or scratches, contamination of mucous membrane with saliva from licks; exposure to bat bites or scratches), without distinguishing the particular epidemiology of rabies in the country of exposure. Category I exposures (touching or feeding animals, licks on the skin) do not require PEP measures.

In this study, we report the epidemiology of rabies in France over a 22-year period from 1995 to 2016, describing and analysing data on human rabies surveillance as well as PEP data collected from the network of French antirabies clinics. Using these data, we elaborate on the need for guidelines to limit overuse of rabies biologics after exposure in rabies-free areas and to support physicians’ efforts to adjust rabies risk assessment, since at the same time the risk of rabies in travellers abroad is increasing.

## Methods

### Human rabies surveillance and diagnosis

Human rabies is a disease that has been subject to mandatory notification in France since 1952. Data from all cases of human rabies diagnosed from 1 January 1995 to 31 December 2016 in France were retrieved from NRCR (database in accordance with the French Act 78–17 on Data Processing, Data Files and Individual Liberties n°1248768, 14/9/2007).

Cases were defined as individuals whose infection was laboratory-confirmed and who were resident in France. The laboratory techniques used for intra-vitam diagnosis of human rabies in the NRCR were those recommended by WHO: heminested and real-time reverse transcription PCR (RT-hnPCR and RT-qPCR, respectively) and virus genotyping by Sanger sequencing in saliva, skin biopsy or cerebrospinal fluid (CSF), and potentially, detection of rabies antibodies by rapid fluorescent focus inhibition test (RFFIT) or ELISA test (Platelia Rabies II Kit, Bio-Rad, Hercules, California, United States (US)) in serum or CSF [25-28]. For post-mortem diagnosis of human rabies, fluorescent antibody test (FAT) was used on brain biopsy as well as rabies tissue culture infectious test (RTCIT) using murine neuroblastoma cells and detection of viral RNA by RT-hnPCR or RT-qPCR [28,29].

Demographic and clinical information about human cases were extracted from notifications and previous publications about each case, if available (demographic data, exposure characteristics, disease presentation and diagnosis methods).

### Post-exposure prophylaxis surveillance

In France, primary healthcare management of individuals seeking care after a potential rabies exposure is delivered through an official national network of 70 clinics distributed throughout the country [23]. Since 1994, 80% to 95% of these centres (depending on year of study) have completed online standardised report forms for all outpatients (Vaccilab/Voozanoo, Epiconcept, Paris, France), accessible only to the members of the network, using individual logins and passwords. Aggregated data concerning PEP are prospectively collected and analysed by the NRCR and published annually [30].

We considered all data concerning individuals seeking PEP in France from 1995 to 2016. Data included patient demographics, animal characteristics, exposure details and information about PEP if administered. The exhaustivity of these data was stable over the study period and estimated to be greater than 90%. Population data from the French Institute of Statistics and Economic Studies [31] were used to calculate the incidence of PEP administration by time period. Data on travel destinations and number of travellers published annually by the French Directorate General for Enterprise were used to analyse PEP travel data and in particular the number of trips outside France made by French residents aged 15 years and older each year [32].

In this study, categorical data are presented as numbers and percentages. Continuous data are presented as median with range (for non-normally distributed data). The chi-squared test was used for categorical data, and a p value of < 0.05 was considered to be statistically significant.

## Results

### Human rabies cases

**Figure 1:**
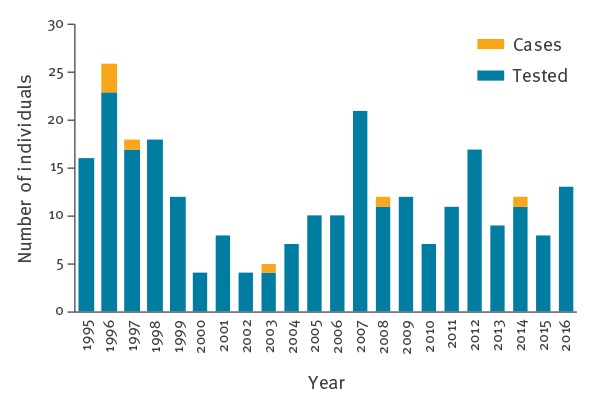
Number of humans tested annually for rabies (n = 250) and laboratory-confirmed human rabies cases (n = 7), France, 1995–2016

During the study period, specimens from 250 individuals with clinically suspected rabies were analysed at the NRCR and seven human cases of rabies were laboratory-confirmed (Figure 1). Six of seven cases were males, only two were children (< 15 years old) and cases’ median age was 50 years (range: 3–71 years) (Table 1). All cases were exposed to rabies abroad, usually following at-risk contact with a dog presumed to be rabid, with the exception of one case who developed the disease in French Guiana and was infected with a virus closely related to those circulating in hematophagous bats in Latin America. For this case, a precise at-risk exposure could not be identified by the epidemiological investigation. No transmission via organ or tissue transplantation was notified in France during the study period. Among the seven cases, only one had PEP administered, without RIG; the remaining cases received no PEP. Duration of incubation period was available for only five cases and ranged from 6 weeks to 2 months, with a median duration of 2 months. For five of seven cases, diagnosis of rabies was made intra-vitam: from skin biopsies (3/5), saliva samples (5/5) and CSF (2/5). For two of the seven cases, diagnosis of rabies was made post-mortem from brain biopsies (Table 1) [25-29]. All rabies-infected cases died after disease duration ranging between 3 and 19 days (median: 7 days).

**Table 1:** Human laboratory-confirmed rabies cases, France, 1995–2016 (n = 7)

Year	Age group (years)	Country of exposure	Animal	Incubation period	Duration of clinical symptoms	Diagnosis intra-vitam	Diagnosis post-mortem	PEP
1996	0-4	Madagascar	Dog	2 months	6 days	NA	FAT and RTCIT on brain sample	No
1996	60-64	Algeria	Dog	2 months	5 days	NA	FAT and RTCIT on brain sample	No
1996	70-74	Algeria	Dog	1.5 months	3 days	RT-PCR on saliva and CSF	NA	No
1997	50-54	India	Dog	12 days	14 days	RT-hnPCR on CSF and saliva	NA	Yes, but no RIG
2003	0-4	Gabon	Dog	2 months	7 days	RT-hnPCR on skin biopsy and saliva	NA	No
2008	40-44	France (French Guiana)	NK	NK	7 days	RT-hnPCR on skin biopsy and saliva	NA	No
2014	55-59	Mali	NK	NK	19 days	RT-hnPCR on skin biopsy and saliva	NA	No

CSF: cerebrospinal fluid; FAT: fluorescent antibody test; NA: not applicable; NK: not known; PEP: post-exposure prophylaxis; RIG: rabies immunoglobulin; RTCIT: rabies tissue culture infection test; RT-hnPCR: heminested and real-time reverse transcription polymerase chain reaction; RT-PCR: real-time reverse transcription polymerase chain reaction.

### Post-exposure prophylaxis

#### Summary of the activities of French antirabies clinics

**Figure 2:**
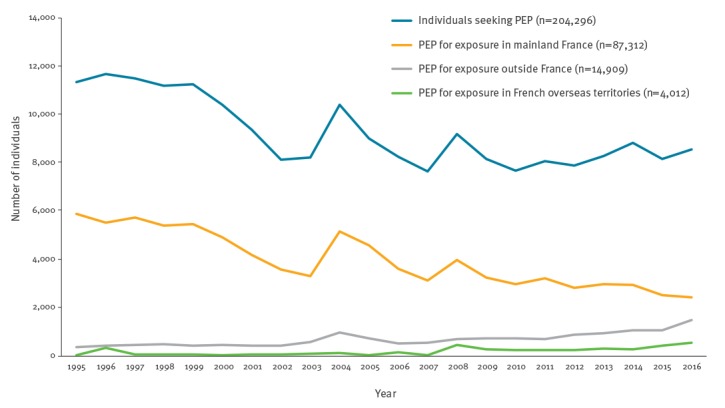
Annual number of individuals seeking post-exposure prophylaxis for rabies (n = 204,296) and post-exposure prophylaxis delivered by location of exposure (n = 106,233), France, 1995–2016

Between 1995 and 2016, 204,296 individuals sought medical attention at an antirabies clinic in France and 106,233 (52.0%) of these received PEP treatment (Figure 2). The median age of individuals receiving PEP was 31 years (range: 0­­–109 years), and 55.4% were male. The annual number of PEP courses delivered in France decreased from 6,254 in 1995 to 4,423 in 2016, representing an incidence of 10.6 PEP per 100,000 population in 1995 and 6.6 PEP per 100,000 population in 2016. PEP incidence peaked in 2004 and to a lesser extent in 2008 and 2011, at the same time as high media coverage of imported rabid animals (Figure 2) [23,33-37].

PEP: post-exposure prophylaxis.

PEP by age group showed a peak for the 20-29-year group, particularly in males (data not shown). The median age of the population receiving PEP tended to rise during the study period, from 27 years in 1995 to 32 years in 2016, as did the median age in the French population, which increased from 35 to 40 years. The monthly distribution of PEP was stable over the study period and showed an increase of vaccine administration in late spring and summer for individuals exposed in France. For travellers exposed outside France, the number of PEP courses administered peaked in July and August (data not shown).

The majority of individuals, 101,538 (95.6%), received purified Vero cell rabies vaccine as PEP, 1,368 (1.3%) received purified chick embryo cell rabies vaccine, 48 and 50 of 106,233 had respectively suckling mouse brain rabies vaccine and human diploid cell culture rabies vaccine. For 3,377 (3.2%) individuals, data concerning the nature of the PEP were missing.

**Figure 3:**
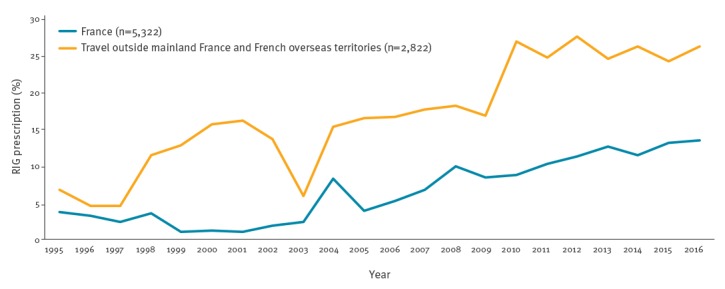
Percentage of prescription of rabies immunoglobulin in individuals given post-exposure prophylaxis, by location of exposure, France, 1995–2016 (n = 8,144)

Among individuals who received PEP, 85,989 (80.9%) had category III exposure (Table 2), 15,723 (14.8%), category II and 2,031 (1.9%), category I [6]. Among individuals with category III exposure, 7,595 (8.8%) were prescribed RIG in France, including 684 (0.8%) who received purified equine immunoglobulin. Individuals exposed abroad were more likely to receive RIG than those exposed in France (20.3% and 6.3% respectively, p < 0.05). However, the proportion of individuals receiving RIG tended to increase in both populations between 2005 and 2016 (Figure 3).

**Table 2:** Characteristics of individuals receiving post-exposure prophylaxis for rabies, by location of exposure, France, 1995–2016 (n = 106,233)

Characteristic	Europe (n = 1,990)		Asia (n = 5,751)		Americas (n = 1,332)		Africa (n = 5,677)		French overseas territories (n = 3,965)		Mainland France (n = 87,518)	
Individuals receiving PEP	n	%	n	%	n	%	n	%	n	%	n	%
Median age in years (range)	33	0–96	31	0–97	31	1–81	32	0–95	29	0–109	31	0–102
< 15 years old	328	16.5 ^a^	742	12.9 ^a^	131	9.8 ^a^	1,466	25.8 ^a^	1,132	28.5 ^a^	18,635	21.3
Male	1,076	54.1	2,813	48.9 ^a^	664	49.8 ^a^	2,922	51.5 ^a^	2,432	61.3 ^a^	48,941	55.9
Category III exposure	1,518	76.3 ^a^	3,654	63.5 ^a^	840	63.1 ^a^	3,439	60.6 ^a^	3,114	78.5 ^a^	73,190	83.6
Dog exposure	1,476	74.2 ^a^	2,793	48.6 ^a^	810	60.8	2,778	48.9 ^a^	2,015	50.8 ^a^	57,073	65.2
Cat exposure	235	11.8 ^a^	582	10.1 ^a^	93	7.0 ^a^	1,487	26.2 ^a^	325	8.2 ^a^	19,343	22.1
Bat exposure	41	2.1	39	0.7 ^a^	83	6.2 ^a^	31	0.5 ^a^	838	21.1 ^a^	1,922	2.2
NHP exposure	64	3.2 ^a^	2,007	34.9 ^a^	93	7.0 ^a^	672	11.8 ^a^	159	4.0 ^a^	336	0.4
RIG	289	14.5 ^a^	1,380	24.0 ^a^	313	23.5 ^a^	1,010	17.8 ^a^	873	22.0 ^a^	4,860	5.6
Proportion of individuals receiving PEP among all PEP-seeking consultations at antirabies clinics	n/N	%	n/N	%	n/N	%	n/N	%	n/N	%	n/N	%
	1,990/2,638	75.4 ^a^	5,751/6,331	90.8 ^a^	1,332/1,520	87.6 ^a^	5,677/6,470	87.7 ^a^	3,965/5,466	72.5 ^a^	87,518/181,871	48.1

NHP: non-human primate; PEP: post-exposure prophylaxis; RIG: rabies immunoglobulin.

^a^ Significantly different from individuals exposed in mainland France (p < 0.05).

RIG: rabies immunoglobulin.

Vaccine tolerability was evaluated for 67,734 individuals (63.8%). Of these, 1,276 (1.9%) experienced systemic side effects, most frequently asthenia and fever, and less frequently nausea, myalgia, cephalgia or allergic side effects. A total of 645 (1.0%) individuals reported local side effects (swelling, redness or pain caused at the injection site) while 65,813 (97.2%) had good tolerance of PEP (data not shown).

Among individuals who received PEP during the study period, 87,518 (82.4%) were exposed in mainland France (free of rabies in non-flying terrestrial mammals but enzootic for bat lyssavirus), 3,550 (3.3%) were exposed in French Guiana (free of rabies in non-flying terrestrial mammals but enzootic for bat rabies virus), 415 (0.4%) in other French overseas territories (free of rabies in non-flying terrestrial mammals and in bats) and 14,750 (13.9%) had exposure abroad in countries potentially enzootic for rabies virus.

#### Exposure in mainland France

**Figure 4:**
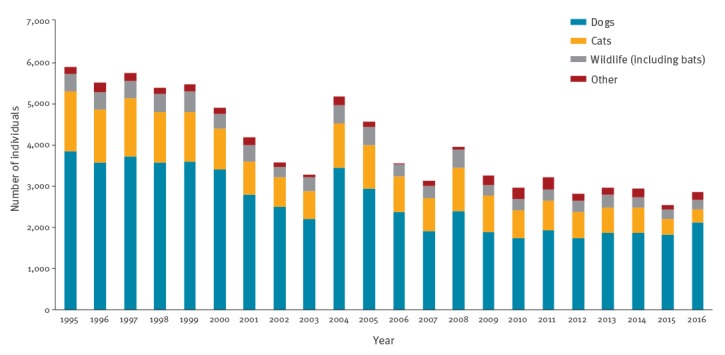
Human exposures leading to rabies post-exposure prophylaxis administration, mainland France, 1995–2016 (n = 57,063)

The cumulative exposure in mainland France to dogs and cats accounted for 57,073 (65.2%) and 19,343 (22.1%) PEP courses, respectively (Figure 4 and Table 2). Wildlife exposure resulted in 7,517 (8.6%) PEP courses, including 1,922 (2.2%) attributed to bats and 336 (0.4%) to non-human primates (NHP). Other types of exposure accounted for 3,631 (4.1%) PEP courses and included exposures to non-identified animals, cattle, laboratory manipulation of virus, manipulation of vaccine baits or exposure to a human rabies case. During the study period, PEP delivered secondary to an exposure in mainland France fell by more than 50%, with an overall proportional decrease in exposure to all animal species (dogs, cats, wildlife and others).

#### Exposure in French overseas territories

From 1995 to 2014, 3,965 PEP courses were delivered following an exposure in French overseas territories, including 3,550 (89.5%) PEP courses following an exposure in French Guiana (Figure 2). The cumulative exposure to dogs, bats and cats accounted for 2,015 (50.8%), 838 (21.1%) and 325 (8.2%) PEP courses, respectively. Compared with individuals exposed in mainland France, those receiving PEP after an exposure in French overseas territories were more likely to be aged under 15 years and male (p < 0.001 and p = 0.003 respectively), and animal exposure was more likely to be due to bats or NHP (p < 0.001) and less frequently to dogs and cats (p < 0.001) (Table 2).

#### Exposure abroad outside mainland France and French overseas territories

**Figure 5:**
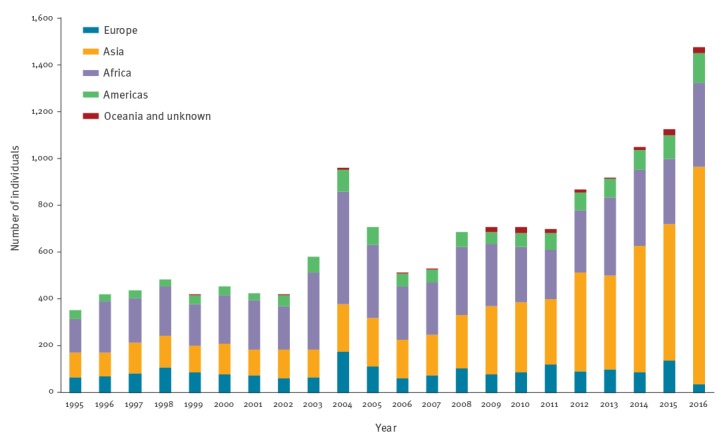
Human exposure abroad outside mainland France and French overseas territories, given post-exposure prophylaxis for potential exposure to rabies virus, France, 1995–2016 (n = 15,000)

Among individuals who received PEP, the number and proportion of exposure that occurred outside mainland France and French overseas territories has increased progressively since 1995, from 354 (5.7%) in 1995 to 1,482 (33.5%) in 2016, and currently represents one third of all PEP courses delivered (Figure 2). In 2016, according to the data published by the French Directorate General for Enterprise, the estimated incidence of PEP courses was 5.5 per 100,000 trips outside France of French residents aged 15 years or older and 0.4, 1.4, 17.9 and 56.7 per 100,000 trips to Europe (not including France), the Americas, Africa and Asia/Oceania, respectively. Exposure occurred in Africa for 5,677 travellers (30.3%) and in particular in three North African countries, with Morocco, Tunisia and Algeria accounting for 66.6% of all travellers to Africa seeking PEP in France. A total of 5,751 (30.7%) travellers were exposed in Asia, 60.1% of whom had been to Thailand, Indonesia or India. Lastly, 1,990 (10.6%) travellers had been to countries in Europe and 1,332 (7.1%) to the Americas. Oceania only accounted for 95 PEP courses (0.5%). During the study period, the destinations of travellers requiring PEP changed, with destinations in Asia becoming more frequently visited than African destinations since the late 2000s. The proportion of individuals exposed in Asia among all PEP administered after travel increased from 29.7% in 1995 to 63.1% in 2016 (Figure 5 and Table 2).

Compared with individuals receiving PEP after an exposure in mainland France, travellers who received PEP were significantly more likely to be female if the exposure occurred in Asia, the Americas or Africa (p < 0.001, p = 0.02 and p < 0.001 respectively). They were likely to be older than 15 years if they had travelled to Europe, Asia or the Americas (p < 0.001) and more likely to be children aged under 15 years if they had travelled to Africa (p < 0.001). People treated after an exposure abroad had significantly less category III exposure (p < 0.001) and received RIG more frequently (p < 0.001). Animal exposures in travellers were mainly caused by dogs, followed by cats and NHP, depending on the traveller’s destination. Compared with the population exposed in mainland France, animal exposures abroad were significantly more likely to be due to dogs and NHP in Europe, NHP in Asia, NHP and bats in the Americas and cats and NHP in Africa (Table 2).

## Discussion

Few countries report exhaustive national data on human rabies deaths and PEP [38]. The organisation of PEP in France allows to collate data efficiently at national level, and so data on French human rabies and PEP could be analysed over a 22-year period.

Human rabies in France is very rare. However, seven cases of human rabies were diagnosed during this period. All but one of them were acquired outside France in countries enzootic for rabies. Six of the seven cases did not seek medical attention, reflecting a lack of awareness about rabies [39-41]. Among all French territories, rabies remains uncontrolled in French Guiana and represents the main hotspot for rabies risk in France. One case developed rabies in French Guiana due to a virus closely related to those circulating in hematophagous bats in Latin America [42,43]. The very low number of human rabies cases during the study period and the absence of autochthonous cases in mainland France mainly reflect the very low risk of being exposed to a rabid animal in France since the declaration of rabies-free status (in non-flying terrestrial mammals) to the World Organisation for Animal Health (OIE) in 2001 [21,44]. However, the low numbers also suggest that the antirabies clinic network is effective on the management of individuals who sought advice on PEP in mainland France before 2001, and of individuals exposed to bats in France or to any animal species in enzootic countries during the study period.

Since France and other western European countries are considered to be rabies-free in non-flying mammals, the main rabies risk in this area results from pets that are imported or travel from rabies-enzootic countries. However, a recent review of rabid animals which focused on pets travelling from a rabies-enzootic country to western Europe calculated an estimated risk of being in contact on a given day with a pet contagious for rabies to be 7.52x10 ^− 10^ [21]. This residual risk is considered to be low by WHO, and negligible by experts from PHE, who do not recommend administering PEP after an exposure to a pet in western Europe [24].

In this regard, it seems that French health professionals are aware of the decreasing risk of rabies in mainland France, as the incidence of rabies PEP has diminished, from 10.6 to 6.6 per 100,000 population from 1995 to 2016. These incidences are below those observed in North America, where rabies is endemic in wildlife (12.1 PEP per 100,000 population reported in 2000 in the state of New York, US [45]; 13.9 PEP per 100,000 population reported between 2001 and 2012 in Ontario, Canada [46]). However, the incidence of PEP in France is still very high compared with the actual risk in western Europe [16,21].

Two major PEP tendencies have been observed over the study period. On the one hand, the incidence of PEP administered following an exposure in France was halved from 1995 to 2016. Of interest, against all expectations and despite the very low rabies risk in France, the proportion of individuals receiving RIG after an exposure in France rose during the study period. In Europe, RIG is mostly derived from human plasma and is a very costly biological product produced in low quantities and associated with recurrent shortages [47]. The over-prescription of PEP and RIG might be partly explained by insufficient knowledge of current rabies epidemiology in France and/or inappropriate information given by first-line healthcare professionals and the difficulty for antirabies clinic physicians in convincing individuals that PEP is not required for all exposures. This over-prescription may also be due to the lack of continuing education for physicians on rabies risk management and, above all, the lack of French guidelines concerning rabies exposure management. Without a national consensus, antirabies clinic physicians currently have to make their own decisions on whether or not to administer PEP [16]. This situation may be improved by the implementation and dissemination of national guidelines.

Although PEP prescribed after exposure in France has decreased, the number of individuals treated with PEP following an exposure abroad has risen in the past 22 years, and now represents one third of all PEP prescribed in France. This tendency might be explained by increased travel abroad, and more frequent at-risk situations while abroad (e.g. outdoor activities, contact with animals) [22]. This situation reflects travellers’ lack of awareness of the threat of rabies in developing countries. The proportion of RIG use after this type of exposure (exposure category and epidemiological circumstances) suggests that most clinicians took into account the epidemiological likelihood that the implicated animal was rabid and followed WHO recommendations [8].

Interestingly, the profile of travel destinations has changed over the study period, with individuals returning from Asia now representing more than half of all those exposed abroad. The need for PEP in individuals returning from Africa has fallen and individuals returning from other destinations in Europe, America and Oceania still represent a minority of those receiving PEP. Individuals receiving PEP and returning from Asia had travelled most frequently to Thailand and Indonesia, which is representative of current travel destination trends for French people [31]. In these countries, NHP and dogs (49.7% and 39.6% of all PEP for these two countries, respectively) dominated animal exposures, in line with the findings of other studies [48].

These results emphasise the increase in travellers among those seeking PEP and highlight the need for recommendations for travellers to help minimise their risk of rabies exposure [49]. Interestingly, six countries (Morocco, Tunisia, Algeria, Thailand, Indonesia and India) accounted for almost half (49%) of all those receiving PEP in France who were exposed abroad. These data are consistent with the results of a recent study evaluating cases of international travellers exposed to potentially rabid animals (the majority of cases being travellers to North Africa or Asia) [22]. Warnings, to be effective, should be targeted to people travelling to the countries identified as having very high risk of exposure.

We recognise two main limitations to our study. First, the PEP data are not fully exhaustive because data reporting to the NRCR is not mandatory and only 80% to 95% of the antirabies clinics reported their data during the study period, depending on the year. However, non-reporting clinics were mainly small centres with a limited number of individuals. The second limitation is linked to the use of a standardised form to report PEP data to the NRCR. This reporting procedure does not allow us to study detailed characteristics of specific exposure situations (e.g. for exposure abroad: reason for travel, duration of stay, whether prophylaxis was initiated abroad).

## Conclusions

Healthcare professionals’ perception of rabies risk is slowly changing in France. The risk of being infected is extremely low or even negligible in mainland France and rabies is now perceived as a threat only if exposure takes place outside western Europe. The decline of PEP administration over the past 22 years illustrates the growing understanding of the changing risk by both the public and healthcare professionals. However, adapting medical practice to the actual rabies risk is a long and difficult process, in the field of potentially lethal and rare communicable diseases, especially in the absence of any active curative treatment.

Information and education are needed at different levels to allay patients’ and healthcare professionals’ unjustified concerns and emphasize real risks. Firstly, healthcare professionals would greatly benefit from validated guidelines at a national or European level to support their efforts to adjust rabies risk assessment to new epidemiological data and to focus on the most important risks: exposure in endemic areas, exposure to imported animals and exposure to bats. Targeted communication about the risk of rabies and preventive measures to travellers to a few countries in Asia and Africa could have a notable effect on the number of PEP courses delivered in western Europe and could contribute to saving lives.
